# Prevalence of self-medication practice among health sciences students in Kermanshah, Iran

**DOI:** 10.1186/s40360-018-0231-4

**Published:** 2018-07-03

**Authors:** Alireza Abdi, Azam Faraji, Fateme Dehghan, Alireza Khatony

**Affiliations:** 1 Department of Nursing, School of Nursing and Midwifery, Kermanshah University of Medical Sciences, Kermanshah, Iran; 2 Social Development and Health Promotion Research Center, Kermanshah University of Medical Sciences, Kermanshah, Iran

**Keywords:** Self-medication, Prevalence, Students

## Abstract

**Background:**

The possibility of self-medication is higher in health sciences students than other students because of easy access to drug information resources and relatively sufficient familiarity with various kinds of drugs. The current study was aimed to determine the prevalence of self-medication and its related factors among the health sciences students.

**Methods:**

A total of 250 health sciences students were included in this cross-sectional study via random sampling. Data were collected by a researcher-made self-medication questionnaire. The collected data were analyzed by SPSS-20 software using descriptive and inferential statistics (chi-square test).

**Results:**

The prevalence of self-medication was 89.6%. Prior experience about the illness, non-seriousness of the illness and availability of drugs were the most prevalent reasons for self-medication. The most commonly used medications included common cold drugs, analgesics and antibiotics. The most frequently used medications were cold pill, acetaminophen pill and amoxicillin capsule. Most students obtained their pharmaceutical information from the pharmacist physician and online sources. Self-medication did not show a significant difference in terms of variables such as age, gender, marital status, insurance status and residence.

**Conclusion:**

Given the high prevalence of self-medication among the health sciences students, training courses about the self-medication risks, more supervision over prohibition of over-the-counter drugs and adequate facilities for students’ access to medical services are suggested to be provided.

**Electronic supplementary material:**

The online version of this article (10.1186/s40360-018-0231-4) contains supplementary material, which is available to authorized users.

## Background

Any arbitrary use of drugs other than prescribed drugs is called self-medication (SM), which can include various kinds of herbal and chemical drugs [1, 2]. The most common reasons for SM include prior experience of the illness, inadequate information about the illness, financial or economic problems for visiting a physician, insufficient time for visiting a doctor and easy access to medications, especially in the developing countries [3]. SM can be dangerous and can cause waste of resources, pharmaceutical reactions and possible increase of antimicrobial resistance [4].

Nowadays, the arbitrary use of over-the-counter drugs among the youth, especially students is on the rise, and the possibility of SM in health sciences students is higher due to easy access to pharmaceutical information resources and relatively adequate familiarity with different kinds of drugs [5]. Numerous studies around the world have analyzed the prevalence of SM among students. In a study carried out among medical and pharmacy students in Jordan in 2016, the prevalence of SM was found to be 78.5% [6].

In another study on 900 health sciences students in Kuwait, the prevalence of SM was reported to be 97.5%, causing headache, menstrual pain and constipation [2]. Further, a study in India showed the prevalence of SM among 440 medical students to be 78.6%. Most of the students reported they used self-medication because of insignificance of illness and having adequate pharmaceutical information, and about half of them used the former prescriptions. The most prevalent medications were antipyretics, antitussives and analgesics. About half of students believed SM was a part of self-care and had to be encouraged [7].

The results of a research conducted on the Brazilian nursing students indicated a prevalence of 76% for SM. The most common reasons for SM were pain, urinary and throat infections and cold. The most prevalent medications used were non-steroid anti-inflammatory drugs [8]. Moreover, a study performed on Iranian health sciences students showed a prevalence of 50.2% for SM, and the most common reason for SM was sufficient information about the illnesses and medications. The most common medications used included antitussives, cold drugs, analgesics and antihistamines. The most common illness lading to SM was reported to be cold [9].

Considering the increasing growth of SM around the world as well as inadequate information about the prevalence of SM among the health sciences students in Kermanshah, the current study was carried out to investigate the prevalence of SM and its related factors among the health sciences students.

## Methods

### Design

This descriptive-analytic study was performed on the medical sciences students of Kermanshah University of Medical Sciences (KUMS), Iran in 2016. This university has six schools, including medicine, pharmacy, dentistry, nursing and midwifery, paramedical sciences and public health, located in the west of Iran.

### Sample

The study sample, based on the results of Ramazani et al. [10] and using the sample size formula for estimating a proportion (95% confidence interval and 0.05 accuracy), was calculated to be 227 students, considered to be 250 students by accounting for 10% chance of loss. Sampling was performed via stratified random sampling method, and sampling classes comprised of the university faculties. According to the number of students in each faculty, a percentage of them in each faculty was selected as study sample. To this end, a list of students in each faculty was first obtained and encoded. Then, using random numbers table, 277 students among different specialties and levels in the faculty were chosen and included in the study. SM in this study was meant the use of any over-the-counter medication over the past 6 months. The inclusion criterion was informed consent for participation in the study.

### Instrument

Data in this study were collected by a two-section questionnaire. The first section was about demographic information such as age, gender, marital status, residence, health insurance, specialty, faculty and monthly income. The second section was a researcher-made questionnaire on SM, which analyzed SM, its causes, illnesses leading to SM, use of medications and information resources of SM. The validity and reliability of the questionnaire had been analyzed and confirmed by Ramazani et al. (α = 0.78) [10] (Additional file 1). The current study also evaluated the validity of the scale by content validity. The questionnaire was given to 12 experts to evaluate, and their corrective comments were applied. To assess the reliability of the questionnaire, Cronbach’s alpha test was used. The questionnaire was given to 30 health sciences students of KUMS to analyze, from which a reliability index of 81% was obtained. It is noteworthy that these students were not included in the study.

### Data collection

To collect the data, the researcher first obtained permission from the Ethical Review Committee of KUMS and then referred to the faculties of university for sampling. To this end, the objectives of the study were first explained to the students and confidentiality of the respondents’ demographic information and their responses was assured. Then, their written informed consent for participation in the study was obtained. Finally, the questionnaires were given to them and collected after completion.

### Data analysis

The obtained data were analyzed by SPSS-22 software using descriptive statistics (frequency, mean and standard deviation) and inferential statistics (chi-square test). To assess the association between SM and demographic characteristics, chi-square test was run. The level of significance was set at *p* < 0.05.

## Results

Among 250 participants in this study, 181 (72.4%) students were female. The mean age of the respondents was 22.07 ± 3.84, 209 (89.3%) of them were single and 218 (87.2%) were under insurance coverage. In terms of academic level, 138 (55.2%) students were undergraduate (BSC), 12 (4.8%) master degree (MSC) and 100 (40%) were Ph.D. As for major, 83 (33.2%) participants were medical students and 65 (26%) of them were studied in paramedical branch. Also, 224 (89.6%) of the sample had a history of SM over the past 6 months prior to the study. The maximum and minimum levels of SM were found for medical disciplines (*n* = 78, 94.1%) and midwifery (*n* = 15, 83.3%). The conditions to run the chi-square test were not met in order to analyze the relationship between SM and major (Table 1).

**Table 1:** Socio-demographic characteristic of respondents by SM

Variables		Total N (%)	Self-medicating N (%)	Non self-medicating N (%)	Statistical tests
Gender	Male	69 (100)	61 (88.4)	8 (11.6)	*X*^2^ = 0.023 *P* = 0.881
	Female	181 (100)	163 (90.1)	18 (9.9)	
Age	18–24	213 (100)	191 (89.7)	22 (10.3)	*X*^2^ = 0.008 *P* = 0.559
	≥ 25	37 (100)	33 (89.2)	4 (10.8)	
Marital states	Single	234 (100)	209 (89.3)	25 (10.7)	*X*^2^ = 0.316 *P* = 0.487
	Married	16 (100)	15 (93.8)	1 (6.2)	
Field of study	Medicine	83 (100)	78 (94.1)	5 (6)	N/A
	Nursing	17 (100)	16 (94)	1 (5.9)	
	Midwifery	18 (100)	15 (83.3)	3 (16.7)	
	Dentistry	11 (100)	10 (90.9)	1 (9.1)	
	Pharmacy	19 (100)	17 (89.5)	2 (10.5)	
	Paramedics	65 (100)	56 (84.8)	10 (15.2)	
	Health	36 (100)	32 (88.9)	4 (11.1)	
Level of graduation	BSC student	138 (100)	121 (87.7)	17 (12.3)	*X*^2^ = 3.373 *P* = 0.185
	MSc student	12 (100)	12 (100)	0 (0.0)	
	PhD student	100 (100)	91 (91)	9 (9.0)	
Location	Living with family	142 (100)	131 (92.3)	11 (7.3)	*X*^2^ = 2.484 *P* = 0.086
	dormitory	108 (100)	93 (86.1)	15 (13.9)	
Insurance	Yes	218 (100)	193 (88.5)	25 (11.5)	*X*^2^ = 2.084 *P* = 0.123
	No	32 (100)	31 (96.6)	1 (3.1)	
Family income (US $)	≤ 307	41 (100)	35 (85.4)	6 (14.6)	N/A
	308–613	96 (100)	87 (90.6)	9 (9.4)	
	≥ 614	111 (100)	101 (91)	10 (9)	

In the current study, the most common illnesses for which SM had been done were common cold (*n* = 187, 74.8%) and headache (*n* = 147, 58.8%) (Table 2). The students reported the most prevalent reasons for SM to be prior experience about the illness (*n* = 145, 58%), none-seriousness of the illness (*n* = 135, 54%) and availability of drugs (*n* = 123, 49.2%) (Table 3). Also, the most prevalent pharmaceutical classes used were common cold drugs (*n* = 145, 58%), analgesics (*n* = 121, 28.8%) and antibiotics (*n* = 109, 43.6%) (Table 4), and the most common drugs used were cold tablets (*n* = 149, 60%), acetaminophen (*n* = 145, 58%) and amoxicillin capsule (*n* = 109, 43.6%).

**Table 2:** Reported diseases/conditions which lead to SM

Disease	N (%)
Common cold	187 (74.8)
Headache	147 (58.8)
Hematological disorders	53 (21.2)
Menstrual disorders^a^	38 (15.2)
Gastrointestinal disorders	40 (16)
Skin diseases	19 (7.6)
Prevention of osteoporosis	17 (6.8)
Musculoskeletal disorders	11 (4.4)
Joint diseases	8 (3.2)
Neurological diseases	7 (2.8)

^a^Only female

**Table 3:** Reasons of SM

Reasons	Number (%)
Prior experience about the illness	145 (58)
Non-seriousness of the illness	135 (54)
Availability	123 (49.2)
Prior experience about the drug	122 (48.8)
Inadequate time to attend the doctor’s office	96 (38.4)
Saving time	95 (38)

**Table 4:** Classes of Drugs used for SM

Class	N (%)
Common cold	145 (58)
Analgesics	121 (48.4)
Antibiotics	109 (43.6)
Vitamins	85 (34)
Anti-allergic	80 (32)
Gastrointestinal drugs	57 (22.8)
Herbal remedies	47 (18.8)
Psychoactive	35 (14)
Tranquilizers	35 (14)
Skin	25 (10)
Antipyretics	25 (10)
Hypnotics	16 (6.4)
Ophthalmic	14 (5.6)

The SM rate was 88.4% among the male students and 90.1% among the female students, indicating no significant difference between them. With regard to marital status, SM was higher in the married students (*n* = 15, 93.8%) than in the single ones (*n* = 209, 89.3%), which was not significantly different. As for residence, the SM rate was higher in the students living with their families (92.3%) than those living in the dormitory (86.1%), showing no significant difference between them. The students with a monthly income more than US $ 614 (*n* = 101, 91%) used more SM than those with a monthly income of US $ 30.8–613 (*n* = 87, 90.6%) and less than US $ 307 (*n* = 35, 85.4%). The data in this part did not have the conditions for running the chi-square test.

The SM rate among the students without medical insurance (*n* = 31, 96.6%) was higher than those with medical insurance (*n* = 193, 88.5%), but the difference was not statistically significant. Pharmacists (*n* = 150, 67%), internet (*n* = 37, 16.5%) and textbooks (*n* = 18, 8%) were the most prevalent sources of information about the drugs used for SM.

## Discussion

The prevalence of SM varies in different countries and regions, ranging from 38.5 to 92% [3, 11–21]. In the present study, the prevalence of SM in the past 6 months prior to the research was about 90%, which is a noticeable rate. The difference in the SM rates may be due to differences in demographic characteristics of the study samples, research methodology, data collection tools and working definition of SM. We tend to think the high prevalence of SM may be due to poor implementation of pharmaceutical rules. In Iran, like many other countries, dangerous and risky medications must be sold with prescription, and even the phrase “not to be sold without prescription” has been labeled on the drug containers. However, this rule is overlooked by many pharmacies due to various reasons like lack of adequate supervision by the concerned authorities.

In the present study, the most prevalent medication categories used were common cold drugs, analgesics and antibiotics. And acetaminophen (*n* = 149, 60%), cold tablet (*n* = 145, 58%) and amoxicillin capsule (*n* = 109, 43.6%) were the most frequently used medications. In various studies conducted on SM, different drug classes have been used. In this regard, the most prevalent pharmaceutical categories used included analgesics [3, 14, 15, 17–19, 21], antipyretics [3, 14, 18, 21], antibiotics [14–16, 18], antihistamines [15], antipruritics [15], and non-steroid anti-inflammatory drugs [19].

Most studies performed on the prevalence of SM have only mentioned the name of pharmaceutical category not the medications of the given category. However, some studies have reported the name of medications in addition to their pharmaceutical class. In these studies, the most commonly used medications are paracetamol [10, 17, 19] and amoxicillin and metronidazole [12]. By taking a quick look at the above drug categories, analgesics and antibiotics are more prominent.

Every medicine has its own side effects, and its arbitrary use can be perilous. For example, some of the side effects of analgesics are hematologic, metabolic, digestive, neural, cardiovascular, hepatic, optic and respiratory complications [22]. This situation is also the same for antibiotics, and arbitrary use of these drugs can be truly disastrous. Antibiotics, even prescribed by the doctors, act like a double-edged sword and can cause unwanted side effects in the patient. The most prevalent side effects of antibiotics are hypersensitivity reactions, anaphylactic reactions, microbial resistance, immune dysfunction and neurologic, renal, cardiac, hematologic and hepatic complications [20, 21]. The authorities of faculties should make use of new training methods like distant education to provide the students with the necessary warnings about arbitrary use of different medications, especially antibiotics [23].

In the current study, the most prevalent reasons for SM in the opinion of students were prior experience about the illness, non-seriousness of the illness and availability of drugs. Numerous studies have reported different reasons for SM by the students, including non-seriousness of the illness [3, 14, 15, 18, 19, 21, 24], prior experience of the illness [14, 19, 21, 24], saving the time [12, 18], availability of drugs [25], past successful use [12, 14], lack of time [14], passing pharmacology course and having sufficient confidence [15], electronic and print media advertisement [25], availability of pharmacist’s consultation [25], lack of access to the health personnel [21] and cost-effectiveness [18], None-seriousness of the illness (mild illness) and prior experience of the illness have been reported in most studies [3, 14, 15, 19, 21, 24].

In the current research, the most prevalent illnesses subjected to SM were common cold and headache. Other studies performed on SM have reported fever [3, 14, 15, 21, 24], headache [3, 14, 15, 17, 19, 21, 24, 25] and cold as the most prevalent illnesses. If SM is carried out by the qualified people who are adequately familiar with the medications, it can be safely used for the treatment of mild diseases like common cold. In this regard, the World Health Organization has accepted SM as a part of self-care process and believes it can reduce the pressure on the healthcare system, especially in areas with limited medical facilities. However, it should be noted that easy access to various medications, especially antibiotics and analgesics can risk the health of individuals and society. Some risks associated with SM include addiction and drug dependence, drug interactions, lower or higher consumption of allowable amount, microbial resistance, and emergence of new microbes and occurrence of different pharmaceutical complications [21]. In this study, SM among the male and female students was not different, which is in line with some studies in this regard [11–13, 24]. However, some studies have reported higher SM in females [19, 25], while some others have shown higher SM in males [15]. We believe SM is a problem that is not associated with gender and is observed in every person, whether a man or a woman.

Moreover, the prevalence of SM was the same in the married and single students. Shah et al. in a study on the students of Karachi University, Pakistan reported similar results [12]. We think SM is a cultural phenomenon that is influenced by various factors, and these factors can get an individual to embark on SM, irrespective of being married or single.

In addition, the findings showed SM was higher among the students living with their families than those living in the dormitory, but the difference was not statistically significant. In our opinion, SM may occur at any place, in the family or in the dormitories.

The students with a monthly income of more than US $ 614 used more SM than those with a monthly income of US $ 308–613 and less than US $ 307, but the difference was not statistically significant. The higher rate of SM among the students with higher income is a surprising issue that needs more analysis.

The findings of this study showed that SM was higher in students without insurance coverage than those under insurance coverage, although the difference was not statistically significant. This finding is in line with the results of Shah et al. [12]. It should be noted that all Iranian students in Iran can have health insurance. However, a significant proportion of students did not use these facilities and had no health insurance. Yet, having or not having insurance has no effect on SM so that the students under health insurance coverage had performed SM as much as the students without health insurance. That students under insurance coverage used SM as much as the students without health insurance is related to the commitments of the insurance companies, and that the insurer should pay a significant portion of the cost of a doctor’s visit and medications.

The most prevalent sources of information about the drugs used in SM were pharmacists and internet. Other studies have reported prior experience [14]. Pharmacist’s advice [3, 14, 15] textbooks [15], classmates or senior students [15] and journals and former prescriptions [3] as the most common sources of information.

Availability of pharmacist’s advice, easy access to pharmaceutical reference books and media advertisement are some reasons for tendency toward SM [25]. In the current study, the highest rate of SM was seen among the medical students. In a study in Ethiopia, no significant difference was found among various majors of health sciences in terms of SM [24], but another study conducted in the same country showed the prevalence of SM to be significantly higher among pharmacy students than other disciplines of health sciences [19].

The studies comparing SM in medical and non-medical students have obtained interesting results. Some of these studies have reported higher prevalence of SM in the medical students than non-medical students [15]. While some others have shown insignificant difference between medical and non-medical students [14, 21]. The results of the present study indicated high prevalence of SM among all health sciences students. This however can be associated with more risks in non-medical students because the medical students have more information about pharmaceutical sciences and have more access to the medical specialists than non-medical students. However, this cannot be a good justification for doing SM by the medical students. On the other hand, the possibility of picking a wrong drug or dosage is higher among the non-medical students.

The current study explored the prevalence of SM in the past 6 months prior to the study. Therefore, there existed the possibility of recall bias. Impossibility of determining the cause and effect relationship due to the cross-sectional nature of this study was another limitation. Because of small sample size, it was not possible to perform chi-square test to investigate the relationship of SM with major and family income. Future studies are suggested to evaluate the prevalence of SM in a larger population and at other medical universities. The predictors of SM are also advised to be analyzed among the health sciences students. Moreover, comparative studies on medical and non-medical students are recommended to be carried out to determine their knowledge about the risks associated with SM.

## Conclusion

In the present study, the prevalence of SM was very high, and SM showed no significant difference in terms of age, gender, marital status, insurance and residence. Considering the risks associated with SM, the authorities of faculties have to provide students with the required trainings in this regard, because the awareness of medical sciences students about SM maybe will reduce its prevalence in general population. Further, more strict policies need to be adopted regarding the promotion and sale of the prescription drugs.

## Additional file

Additional file 1: Questionnaire. The questionnaire of self-medication to assess the prevalence of self-medication in student. The tool has 16 questions which include demographic information and some details about self-medication, causes and medications. (DOCX 14 kb).

## Abbreviations

KUMS: Kermanshah University of Medical Sciences
SM: Self-medication
