# Supplementary material for: Prevalence of self-medication practice among health sciences students in Kermanshah, Iran
Source: BMC Pharmacol Toxicol. 2018 Jul 3;19:36. doi: 10.1186/s40360-018-0231-4 (PMC6029137; doi:10.1186/s40360-018-0231-4)
Supplement: Supplementary file 1 — Questionnaire. The questionnaire of self-medication to assess the prevalence of self-medication in student. The tool has 16 questions which include demographic information and some details about self-medication, causes and medications. (DOCX 14 kb). [file 40360_2018_231_MOESM1_ESM.docx]

Dear student

Regarding the importance of the students’ role in societies, this questionnaire has been provided to assess the arbitrary use of medication (self-medication). Your precise response can promotes the society health. It is notable there is no need to write your name and the right of confidentiality will be respected.

1. Age:…..year
2. Sex: male …… female…..
3. Educational level: BSC….. MSc ……. PhD……
4. Educational school: medicine….. pharmacy …… paramedical…… nursing and midwifery…… health……. Dentistry………
5. Educational field: medicine……. Nursing……. Midwifery…… dentistry…….

Pharmacy …… paramedics…….. Health……..

1. Marital status: married….. Single…….
2. Live location: urban…… rural…….
3. Habitation type: with family ……. student dormitory ……..
4. Family income: ………. Tomans
5. Covered by health insurance yes…… no………
6. Which insurance?
7. The sources for taking your medication information: physicians …… family and friends…….. books/booklets…….. Journals …….. Radio …… TV…… internet…… other…….
8. Did you do self-medication during previous 6 months? Yes no
9. If yes, about which disease?

| **Disease** | **yes** | **no** |
| --- | --- | --- |
| Common cold |  |  |
| headache |  |  |
| Hematological disorders |  |  |
| Menstrual disorders |  |  |
| Gastrointestinal disorders |  |  |
| Skin diseases |  |  |
| Prevention of osteoporosis |  |  |
| Musculoskeletal disorders |  |  |
| Joint diseases |  |  |
| Neurological diseases |  |  |
| Other | Name of disease | |

1. During previous 6 months which of the medication did you use?

| **Class** | **yes** | **no** |
| --- | --- | --- |
| Common cold |  |  |
| Analgesics |  |  |
| Antibiotics |  |  |
| Vitamins |  |  |
| Anti-allergic |  |  |
| Gastrointestinal drugs |  |  |
| Herbal remedies |  |  |
| Psychoactive |  |  |
| Tranquilizers |  |  |
| skin |  |  |
| Antipyretics |  |  |
| Hypnotics |  |  |
| Ophthalmic |  |  |

1. What were the main reasons of your self-medication?

| **Reasons** | **yes** | **no** |
| --- | --- | --- |
| Prior experience about the illness |  |  |
| Non-seriousness of the illness |  |  |
| Availability |  |  |
| Prior experience about the drug |  |  |
| Inadequate time to attend the doctor’s office |  |  |
| Saving time |  |  |
| other | With name | |
